# Dabrafenib plus trametinib in an elderly patient with BRAF V600E-mutant advanced pancreatic adenocarcinoma: A case report

**DOI:** 10.3389/fonc.2025.1687796

**Published:** 2025-11-24

**Authors:** Linger Liu, Xiaolian Zhu, Yinhong Guo, Mengyao Tang, Wu Zhou, Baisong Chen

**Affiliations:** 1 Department of Oncology, Zhuji People’s Hospital of Zhejiang Province, Zhuji, Zhejiang, China; 2 Department of Internal Medicine, School of Medicine, Shaoxing University, Shaoxing, Zhejiang, China

**Keywords:** pancreatic adenocarcinoma, BRAF V600E, case report, dabrafenib, trametinib, dose-adjusted

## Abstract

Despite the success of anti-*BRAF* therapy in melanoma, data from randomized clinical trials are lacking for targeted therapy against *BRAF* mutations—typically the V600E variant—in pancreatic adenocarcinoma, which is associated with a poor prognosis under traditional cytotoxic chemotherapy. Here, we report a case of an elderly patient with advanced pancreatic adenocarcinoma harboring a *BRAF* V600E mutation who received low-dose dabrafenib and trametinib and achieved satisfactory clinical outcomes. We describe a 78-year-old female with *BRAF* V600E-mutant pancreatic adenocarcinoma. The patient was diagnosed with AJCC clinical stage IV (cT3N2M1) pancreatic adenocarcinoma and she declined chemotherapy because of her advanced age. Owing to the *BRAF* V600E mutation, the patient was started on combined *BRAF*- and *MEK* inhibitors (dabrafenib/trametinib). CT scans showed PR on 31 December 31, 2024, and repeated CT scans showed SD on May 2025. At the time of drafting this report, the patient had achieved 8 months of PFS. This case suggests that dose-adjusted dabrafenib combined with trametinib might be a potentially effective treatment strategy for elderly patients with advanced pancreatic adenocarcinoma harboring *BRAF* V600E mutations.

## Introduction

Pancreatic adenocarcinoma (PAC) represents 90% of all pancreatic cancers and is aggressive with a poor prognosis (1). It is estimated to become the second leading cause of cancer-related death in the United States by 2030, with a 5-year survival rate of about 10% (2, 3). Since the pancreas is located in the retroperitoneum, the disease often manifests insidiously with nonspecific symptoms. Most patients diagnosed at advanced stages are unable to undergo surgical resection (4).

The first-line chemotherapy treatments for advanced disease are AG (gemcitabine and nab-paclitaxel) and FOLFIRINOX (oxaliplatin, irinotecan, fluorouracil, and leucovorin). However, the median overall survival (OS) for these two regimens is 8.7 months (as reported by Goldstein D, et al.) and 11.1 months (as reported by Conroy T, et al.), respectively (5–7). In the phase III NAPOLI 3 trial, first-line NALIRIFOX (liposomal irinotecan, fluorouracil, leucovorin, and oxaliplatin) significantly improved median OS compared with AG (11.1 *vs* 9.2 months; HR 0.83, p = 0.036) in metastatic pancreatic ductal adenocarcinoma. The efficacy difference between the NALIRIFOX regimen and the FOLFIRINOX regimen remains to be elucidated (8). Due to the propensity for resistance to currently available therapies, more effective treatment strategies for patients with advanced pancreatic adenocarcinoma are needed.

*BRAF* mutations, typically V600E, can activate downstream kinases and culminate in uncontrolled cell growth and survival (9). The combination of dabrafenib (a *BRAF* inhibitor) and trametinib (a *MEK* inhibitor) has been approved by the FDA for treating advanced or metastatic melanoma, non-small cell lung cancer, and anaplastic thyroid cancer with *BRAF* V600E mutations, with reported objective response rates (ORRs) of 64%, 38%, and 56%, respectively (10–13). However, the efficacy of targeting the same genetic alteration varies across different tumors (14).

In pancreatic adenocarcinoma, about 3% of patients harbor the *BRAF* V600E mutations (15). Due to the rarity of these mutations, reported research on *BRAF* inhibitors in pancreatic adenocarcinoma is restricted primarily to case reports. Here, we add a case to the growing literature describing an elderly patient with advanced pancreatic adenocarcinoma harboring a *BRAF* V600E mutation who received low-dose dabrafenib combined with trametinib and achieved clinical benefit.

## Case presentation

**Figure 1:**
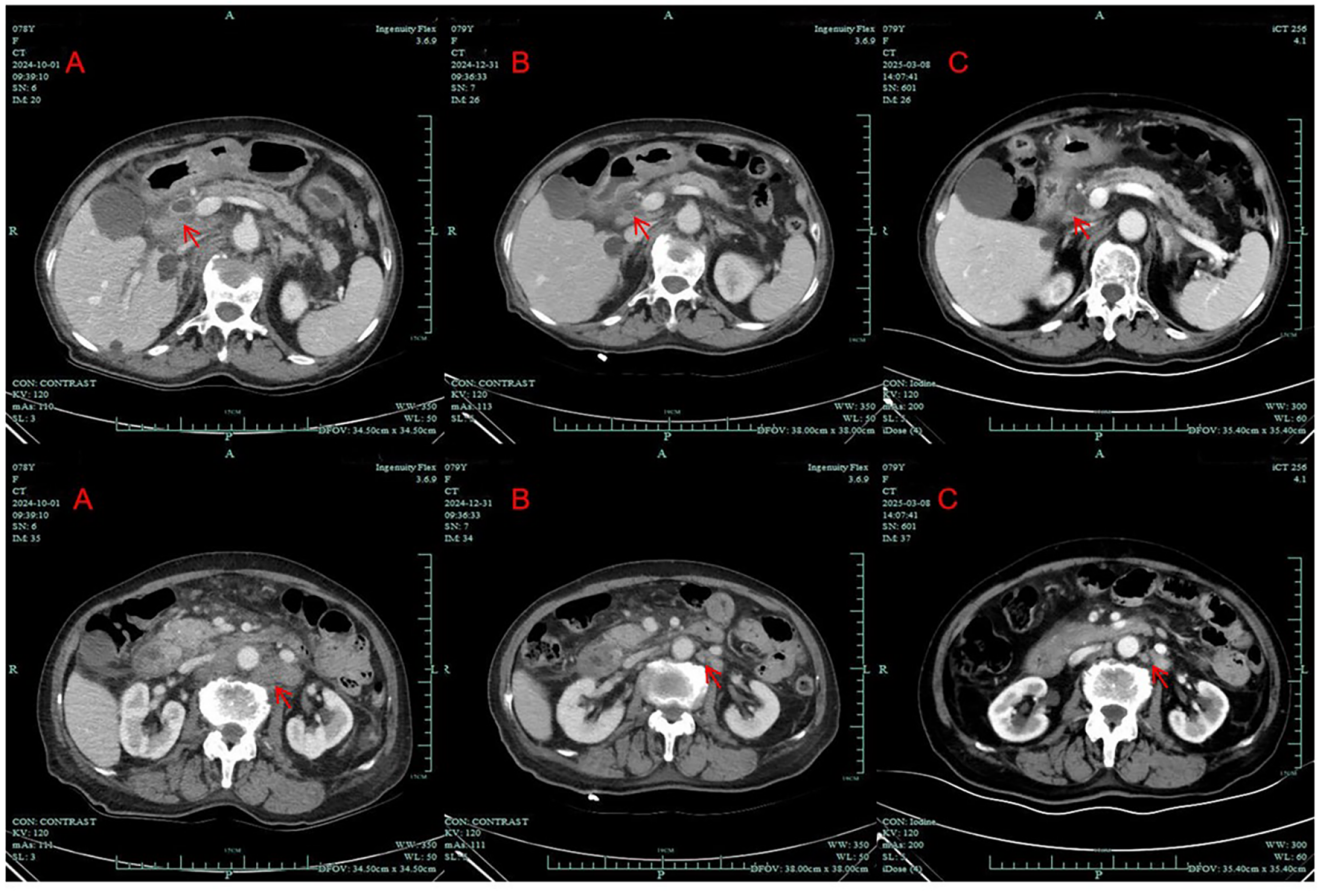
Computed tomography scans of the patient; **(A)** before treatment (October 1, 2024); **(B)** after 7 weeks of treatment with dabrafenib and trametinib (December 31, 2024); **(C)** after approximately 4 months of treatment with dabrafenib and trametinib (March 8, 2025). (arrows: tumor lesion and enlarged lymph node).

A 78-year-old Chinese female who initially presented with back pain was admitted to our hospital (Zhuji People’s Hospital) on September 2024. On October 2024, contrast-enhanced computed tomography (CT) images showed enlargement of the pancreatic head, dilation of the pancreatic and bile ducts, and swelling of multiple peripancreatic and retroperitoneal lymph nodes (**Figure 1A**). A positron emission tomography/computed tomography (PET/CT) scan indicated the possibility of a malignant tumor originating from the pancreatic head, with the enlarged lymph nodes in the peripancreatic, hepatic portal, portocaval, hepatogastric, retrocrural, retroperitoneal, bilateral iliac, and bilateral inguinal regions, consistent with metastatic disease. The CA19–9 level was 757 KIU/L (normal reference value range: 0.0–30.0 KIU/L). Her medical history included hypertension for 10 years, controlled with valsartan and felodipine. She was a non-smoker and had a height of 160 cm and a weight of 52 kg. On physical examination, neither the spleen nor the liver was palpable. No family history of cancer was noted.

**Figure 2:**
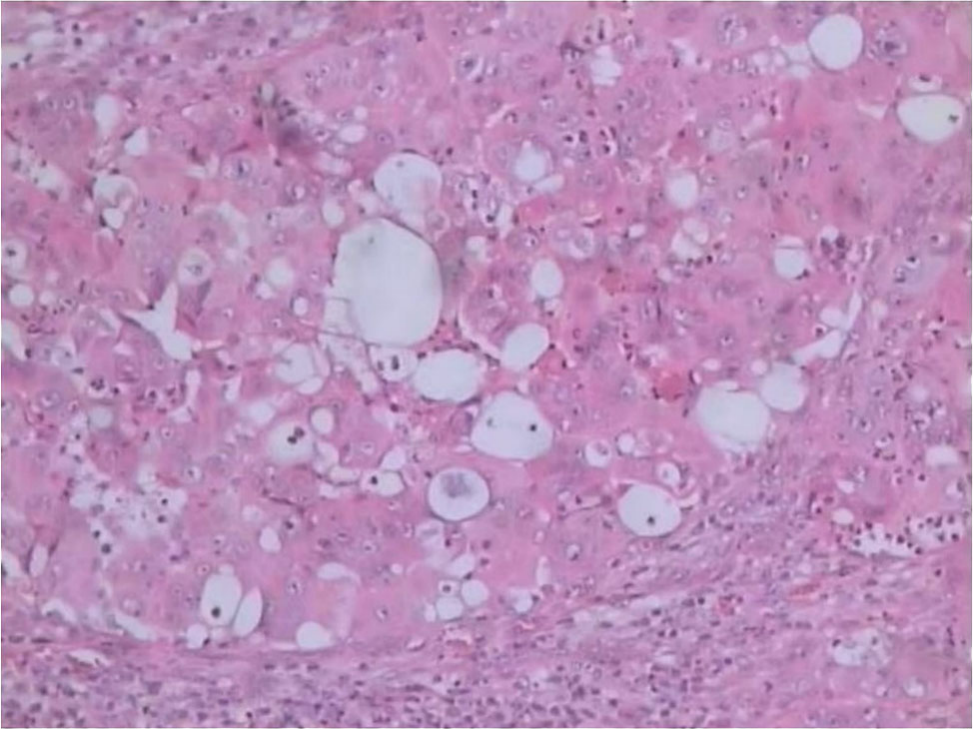
Histopathological image of metastatic pancreatic adenocarcinoma (hematoxylin-eosin, original magnification x100).

The patient underwent left inguinal lymphadenectomy on 7 October 7, 2024, which revealed a mass in the inguinal region measuring 2.0 × 2.0 cm. Pathological examination confirmed metastatic adenocarcinoma consistent with pancreatic cancer (**Figure 2**). The Ki-67 index was 70%. Genetic testing identified a *BRAF* V600E mutation and a TP53 mutation and revealed wild-type status for *ALK, BRCA1/2, PIK3CA, EGFR, ERBB2, KRAS, NRAS*, and *ROS1*. The patient was diagnosed with clinical stage IV (cT3N2M1) pancreatic adenocarcinoma according to the American Joint Committee on Cancer 8th edition staging system.).

The patient received two cycles of chemotherapy with gemcitabine plus nab-paclitaxel on October 2024 and November 2024, respectively. However, she did not complete the full course of the second chemotherapy cycle due to personal reasons. Considering her age, she and her family declined further chemotherapy. At that time, the CA19–9 level was 778 KIU/L. Because of the presence of the *BRAF* V600E mutation and the patient’s advanced age, she received oral low-dose dabrafenib (50 mg twice daily) and trametinib (2 mg once daily) with informed consent from November 2024.

CT imaging assessment was conducted on 31 December 31, 2024. The best response according to the Response Evaluation Criteria in Solid Tumors (RECIST) 1.1 was partial response (PR) (**Figure 1B**). The CA19–9 level decreased steadily. Repeated abdominal CT scans showed stable disease (SD) on 8 March 8, 2025 (**Figure 1C**). The CA19–9 level was 192.0 KIU/L at that time. Repeated CT scans still showed SD on 26 May 26, 2025.

In addition, the patient tolerated treatment with dabrafenib and trametinib well. No grade 3 or 4 treatment-related adverse events (TRAEs) were observed during the treatment period. At the time of drafting this case report, the patient had achieved 8 months of PFS (progression-free survival (PFS).

## Discussion

Pancreatic adenocarcinoma is often diagnosed late at advanced stages and traditionally portends a dismal prognosis. Currently, genomic sequencing enables approaches for molecularly targeted therapies, but few effective targeted therapies have been confirmed in PAC (16). There are four known major known gene mutations in pancreatic adenocarcinoma: *KRAS, TP53, CDKN2A*, and SMAD4. However, none of these has been effectively targeted in clinical practice using current therapeutic regimens (17, 18).

The only targeted agent currently approved for pancreatic adenocarcinoma now is olaparib for patients harboring *BRCA* 1/2 mutations, but these mutations are present in only 5% of patients with pancreatic cancer (19, 20). Due to the lack of effective treatments for common mutations, we believe that the precision therapies for subsets of patients with specific genetic alterations are the key to advancing treatment strategies for advanced pancreatic adenocarcinoma.

The *RAS/RAF/MEK/ERK* pathway, also known as the mitogen-activated protein kinase (MAPK) pathway, is a key intracellular signaling pathway that regulates diverse cellular functions and plays a vital role in oncogenesis and the growth of transformed cells (13, 21). *BRAF*, a serine/threonine kinase located immediately downstream in the Ras signaling pathway, is mutated in approximately 15% of all cancers (22). When mutated, *BRAF* can activate downstream kinases and culminate in uncontrolled cell growth and survival (9). Most mutations arise from the substitution of valine with glutamic acid at codon 600, known as the *BRAF* V600E mutations. These mutations were reported by Yaman B et al. to show an inconsistency rate of up to 14.5% between primary and metastatic lesions in melanoma cases, although analogous analyses have not been conducted in pancreatic cancer (23).

*BRAF* V600E mutations are present in about half of melanomas and have been successfully targeted with *BRAF* inhibitors in melanomas, such as dabrafenib (24). It is worth noting that the combination of *BRAF* inhibitors and *MEK* inhibitors, such as trametinib—which suppresses *MEK1/2* and thereby blocks downstream signaling of the MAPK pathway—has been introduced to reduce hyperproliferative cutaneous events and delay the development of acquired drug resistance during *BRAF* monotherapy. Thus, the combination of *BRAF* and *MEK* inhibitors has become the standard treatment for advanced melanoma (25).

Research has shown that the *BRAF* V600E mutation is mutually exclusive with *KRAS* mutation (9). Among patients with *KRAS* wild-type pancreatic adenocarcinoma (representing 10% of all cases), 30% harbor *BRAF* mutations, accounting for 3% of all PAC. To date, no therapeutic trials have been published targeting this rare molecular subgroup. These mutations are typically associated with poor prognosis (15, 26, 27). A phase II trial (NCT04390243) is currently underway to assess the efficacy of the combination therapy with binimetinib and encorafenib in pancreatic cancer patients with with a somatic *BRAF* V600E mutation (28). Apart from this, reported research on *BRAF* inhibitors in *BRAF*-mutated pancreatic adenocarcinoma is limited to a handful of case reports or brief mentions within larger analyses of all non-melanoma cancers. We summarize the relevant cases of advanced pancreatic adenocarcinoma patients treated with *BRAF* inhibitors in **Table 1**. For example, Grinshpun et al. reported that a patient with advanced pancreatic adenocarcinoma and *BRAF* V600E mutation who received treatment with dabrafenib plus trametinib, resulting in a marked decline in CA19–9 levels; however, the patient died of an acute abdomen after only 19 days of treatment (29). Sasankan et al. reported on a 49-year-old patient with pancreatic adenocarcinoma harboring a *BRAF* V600E mutation who was treated with dabrafenib and trametinib as second-line therapy. The dosages of both agents were reduced due to treatment-related toxicity, including septic shock and neutropenic fever. The patient responded well for 8 months before experiencing progressive disease (PD) (14). However, due to the small number of reported cases, it remains difficult to draw definitive conclusions.

**Table 1:** BRAF inhibitor therapy in pancreatic adenocarcinoma: a brief literature review.

Authors year	Patient	Mutation type	Treatment	Targeted therapy outcomes
Busch et al. 2018 (30)	27 F	BRAF V600E	FOLFIRINOX DT	PFS: Not mentioned OS: 21 months
Aguirre et al. 2018 (31)	66 F	BRAF N486-P490 deletion	FOLFIRINOX AG Trametinib Ulixertinib/BVD-523	PFS (Trametinib): 6 months OS: ~15 months
Kazimierz O et al. 2019 (32)	65 M	BRAF ΔN486_P490 variant	AG Folfiri Dabrafenib	PFS: 6 months OS: N/A
Grinshpun A et al. 2019 (29)	75 F	BRAF V600E	Oxaliplatin-based chemotherapy DT	PFS: 19 days OS: ~1 month
Grinshpun A et al. 2019 (29)	56 M	BRAF c1799_1801delTGA	Gemcitabine DT	PFS: 3 months OS: 6 months
Cramer et al. 2020 (33)	15 F	BRAF V600E	AG DT	CR for 24 months PFS: N/A OS: N/A
Sasankan et al. 2020 (14)	49 F	BRAF V600E	AG DT	PFS: 8 months OS: N/A
Seghers et al., 2020 (34)	66 M	BRAF V600E	AG Vemurafenib/cobimetinib	PFS: 9 months OS: N/A
Shin et al. 2020 (10)	83 M	BRAF NVTAP deletion	AG DT	PFS: 6 months OS: 6 months
Ardalan et al. 2021 (35)	60 F	BRAF V600E	FOLFIRINOX AG AG/Cobimetinib	CR for 6 months PFS: N/A OS: N/A
Li et al. 2022 (26)	34 M	BRAF V600E	Nab-paclitaxel/Oxaliplatin AG DT	PFS: 12months OS: N/A
Wang et al. 2022 (24)	66 M	BRAF V600E	AG Vemurafenib/Trametinib Vemurafenib/Cobimetinb	PFS: 17 months OS: N/A
SHIVANI SHAH et al. 2023 (5)	75F	BRAF V600E	FOLFIRINOX DT	CR for 7 months PFS: N/A OS: N/A
SHIVANI SHAH et al. 2023 (5)	81 M	BRAF V600E	AG mFOLFIRINOX DT	SD for 9 months PFS: N/A OS: N/A

F, female; M, male; DT, dabrafenib plus trametinib.

In this report, we share a case of a 78-year-old patient with advanced pancreatic adenocarcinoma who refused chemotherapy due to advanced age and subsequently received low-dose dabrafenib combined with trametinib based on the presence of a *BRAF* V600E mutation. She tolerated the modified doses well, and repeat CT scans after 7 weeks of treatment showed PR. At the time of drafting this report, she had achieved 8 months of PFS. The patient is still being monitored for further response.

Because of her advanced age and concerns regarding adverse reactions, the patient initiated treatment with a lower dose of dabrafenib (50 mg, twice daily), which is below the reduced dosages reported in other case reports (e.g., 150 mg twice daily). The patient has demonstrated a non-inferior survival benefit compared with previously reported cases and showed favorable tolerability. In view of the successful experience of this case, we suggest that dose-adjusted dabrafenib plus trametinib might be a potentially effective treatment strategy for elderly patients with advanced pancreatic adenocarcinoma harboring *BRAF* V600E mutations. We aim to add a new case to the available literature with the hope of contributing to the growing discussion regarding the treatment of advanced pancreatic adenocarcinoma with *BRAF* mutations.

## Conclusions

We report a case of an elderly patient with *BRAF* V600E-mutant advanced pancreatic adenocarcinoma who received low-dose dabrafenib plus trametinib and achieved satisfactory clinical outcomes. Dose-adjusted dabrafenib combined with trametinib might be a potentially effective treatment strategy for elderly patients with advanced pancreatic adenocarcinoma harboring *BRAF* V600E mutations, and needs to be further evaluated clinically.

## Data Availability

The raw data supporting the conclusions of this article will be made available by the authors, without undue reservation.
